# Social behaviour mediates the microbiome response to antibiotic treatment in a wild mammal

**DOI:** 10.1098/rspb.2024.1756

**Published:** 2024-10-02

**Authors:** Bianca R. P. Brown, Allison E. Williams, Kate A. Sabey, Aaron Onserio, John Ewoi, Se Jin Song, Rob Knight, Vanessa O. Ezenwa

**Affiliations:** ^1^ Department of Ecology and Evolutionary Biology, Yale University, New Haven, CT, USA; ^2^ Odum School of Ecology, University of Georgia, Athens, GA, USA; ^3^ Department of Infectious Diseases, College of Veterinary Medicine, University of Georgia, Athens, GA, USA; ^4^ Mpala Research Centre, Nanyuki, Kenya; ^5^ Center for Microbiome Innovation, University of California San Diego, La Jolla, CA, USA; ^6^ Department of Pediatrics, University of California San Diego, La Jolla, CA, USA; ^7^ Department of Computer Science and Engineering, University of California San Diego, La Jolla, CA, USA; ^8^ Department of Bioengineering, University of California San Diego, La Jolla, CA, USA

**Keywords:** 16S rRNA, degree, social connectivity, gazelle

## Abstract

High levels of social connectivity among group-living animals have been hypothesized to benefit individuals by creating opportunities to rapidly reseed the microbiome and maintain stability against disruption. We tested this hypothesis by perturbing the microbiome of a wild population of Grant’s gazelles with an antibiotic and asking whether microbiome recovery differs between individuals with high versus low levels of social connectivity. We found that after treatment, individuals with high social connectivity experienced a faster increase in microbiome richness than less socially connected individuals. Unexpectedly, the rapid increase in microbiome richness of highly connected individuals that received treatment led to their microbiomes becoming more distinct relative to the background population. Our results suggest that the microbiome of individuals with high social connectivity can be rapidly recolonized after a perturbation event, but this leads to a microbiome that is more distinct from, rather than more similar to the unperturbed state. This work provides new insight into the role of social interactions in shaping the microbiome.

## 1. Introduction

Social interactions are a key driver of pathogen transmission in animal populations [1]. Recently, it has been shown that social behaviour also plays a significant role in the transmission of commensal gut microbes [2–6]. Among group-living animals, social interactions can create opportunities for horizontal microbiome transmission through direct contact (e.g. physical interaction) or indirect contact (e.g. shared space use) [7,8]. Since the gut microbiome provides a plethora of critical functions to its host, such as protection against pathogens and extraction and absorption of nutrients, access to a potentially greater diversity of commensal bacteria could be considered a benefit of group living [9–11]. It is also hypothesized that social transmission of commensal microbes can result in more stable and resilient individual microbiomes because group members serve as sources for replenishing the microbiome during perturbation events [8]. Given substantial heterogeneity among individuals in the frequency and extent of social interactions, this hypothesis implies that individuals with more social connections should derive greater microbiome-related benefits, i.e. their microbiomes should recover faster from perturbation.

Wild animal populations are increasingly exposed to anthropogenic threats that perturb their microbiome. For example, the expanding use of antibiotics to battle human and veterinary animal pathogens has resulted in antibiotic spillover into wildlife species, reflected in an increased number of antibiotic-resistant genes in wild microbiomes [12–14]. Yet, antibiotic-induced microbiome changes are rarely studied in wild animals despite there being demonstrable consequences for humans and domestic animals [15,16]. In this context, quantifying the influence of social behaviour on microbiome recovery can contribute to a general understanding of factors that facilitate the microbiome stability of wild animals in the face of antibiotic disruption.

Here, we investigated how variation in social connectivity influenced gut microbiota recovery after antibiotic perturbation in a wild population of female Grant’s gazelle (*Nanger granti*). Grant’s gazelles are polygynous ungulates with a resource defence-based mating system in which males defend resource patches (i.e. territories) that vary in quality, and females track resources by selecting among male territories [17]. This results in a fluid social system in which females frequently move between social groups, with variation in social behaviour determined by individual life history and environmental context [18]. As such, social interactions among adult female gazelles are primarily characterized by indirect contact through shared space use. By tracking individual gut microbiome richness and composition after antibiotic treatment of female gazelles, we tested the hypothesis that more socially connected individuals recover more quickly from microbiome perturbation. Specifically, if social interactions create opportunities for microbiome transmission, we predicted that following antibiotic treatment, more socially connected individuals would show: (i) faster increases in microbiome richness and (ii) closer microbiome resemblance to untreated individuals over time.

## 2. Methods

### (a) Animal capture and treatment

We captured and sampled 51 female gazelles at Mpala Research Centre (MRC), Kenya over a four day period in June 2015 using handheld net guns fired from a helicopter. Study animals ranged in age from 0.5 to 10 years of age, with a mean of 6 years (±2.5 years). All animals were given unique colour ear tags and randomly assigned to an antibiotic treatment or control group based on the sequence of capture. Half of the individuals (treated) were given a single intramuscular injection of oxytetracycline (20 mg kg^−1^) and the other half (control) received saline injections. Oxytetracycline is a broad-spectrum antibiotic commonly used in livestock that is effective against gram positive and negative bacteria [19,20]. The one-time application perturbed the gazelle microbiome over both short (≤30 days) and longer (90 days) time scales (Sabey *et al*. *in review* [21]).

### (b) Behavioural observations and social network analysis

Grant’s gazelle groups typically comprise either a single territorial male with adult females and juveniles or multiple non-territorial males [17]. At MRC, the size of female groups within a territory can range from 2 to 20 individuals and females frequently move among territories [18]. To quantify individual social connectivity in females, we monitored treated and control individuals for 90 days following capture. We located groups by driving regular road transects between 06.30 and 18.30 h, defining a group as a spatially distinct set of individuals engaged in coordinated activity [18].

Once a group of animals was sighted, we recorded the size, composition and location (GPS coordinate) of the group as well as the identity of all tagged individuals. We observed 51 tagged individuals between 3 and 52 times during the study period. We used these data to construct a pairwise social association matrix for the entire 90 day study period following the ‘gambit of the group’ assumption, where any two individuals observed in the same group were considered to be associated [22]. We quantified associations on a daily scale, so individuals had the potential to be associated between 0 and 90 times throughout the study. Next, we used the association matrix to generate a social network using the *asnipe* package in R [23]. In the network, individuals are represented by nodes and edges are the associations between nodes. Edges were estimated using the half weight index (HWI), which corrects for biases introduced by missed sightings of focal individuals, providing a closer estimate of the real rate of association [24]. One limitation of the HWI is that it assumes all individuals have equal opportunities for interaction [25]. We used the R package *sna* [26] to calculate weighted degree as a measure of social connectivity. Weighted degree (hereafter called degree) is defined as the total sum and weight (i.e. number of reoccurring interactions) of edges connected to a node [23]. Since animal identities were unknown prior to animal capture and tagging, degree was only estimated after, not before, treatment.

### (c) Microbiome sampling, processing and sequencing

We characterized the gut microbiome using faecal samples collected during behavioural observations. When a tagged individual was observed defecating, the sample was collected within 10 min, placed in a sterile 2 ml tube and stored on ice in the field. In the lab, samples were stored at −20°C until further processing. Microbiome samples were collected from 32 (12 control, 20 treated) of 51 individuals contributing to the social network for a total of 91 samples, including 38 from control animals (mean and range per individual: 3 [1–5]) and 53 from antibiotic-treated animals (mean and range: 3 [1–7]).

Total DNA was extracted from all samples using the MoBio PowerSoil DNA extraction kit. Amplification and sequencing of samples and pre-processing of data were performed following Earth Microbiome Project protocols [27]. Briefly, we targeted the microbiome by amplifying the V4 region of the 16S rRNA bacterial gene primers 515F and 806R in triplicate. Amplicons were sequenced on an Illumina MiSeq (2 × 150 bp). We removed sequence reads of less than 150 bp in length and used Deblur (v. 1.1.0) [28] to cluster reads into amplicon sequence variants (ASVs) that were imported to *QIIME2* for analysis. We built a phylogenetic tree based on ASVs and assigned taxonomy using the Greengenes 2 reference database in *QIIME2* [29]. To remove ASVs corresponding to mitochondria and chloroplasts, we assigned taxonomy using the Greengenes database (v. 13.8) as described in Sabey *et al*. [21, *in review*].

We also removed ASVs with a relative abundance of less than 0.01% across all samples. Following the filtering steps, all samples were rarified to 7000 reads, and our final taxa table consisted of 979 unique ASVs. To assess phylogenetic relationships among ASVs, we used the reference phylogeny from the Greengenes 2 database (v. 2022.10 [30]).

### (d) Statistical analyses

To examine whether the rate of gain in microbial richness after treatment depended on social connectivity, we used linear mixed effects models (LMMs). We used *QIIME2* to estimate different dimensions of alpha diversity quantified as observed richness, Chao 1, and Shannon diversity. Richness estimates the total number of ASVs without considering abundance; Chao1 better captures the presence of rare ASVs and Shannon diversity combines richness and evenness [31], better reflecting changes in ASV proportions. We ran separate LMMs for control and treated individuals post-treatment, with each diversity metric used as a response variable and social connectivity (degree), age (in years), time (days post-treatment), and the interaction between degree and time as predictor variables. We chose to run separate LMMs by treatment group rather than include a three-way interaction in a single model (degree × treatment × time) to reduce model complexity and facilitate model interpretation. For all models, animal ID was included as a random intercept to account for repeated sampling of individuals. LMMs were run using the *lme4* package [32] in R and model validity was evaluated via inspection of residuals as described in Zuur *et al.* [33]. In addition, for models showing significant (*p *< 0.05) or marginal (*p *< 0.059) effects of social connectivity, we performed influence tests using the *influence.ME* package [34]. We used animal ID as the grouping level for the influence analyses since this was the level used for the random intercept in all LMMs, and we identified influential observations using a common Cook’s distance cut-off (Cook’s *D* > 4/*n*), where *n* is the number of observations in the analysis [34]. All animal IDs with influence estimates higher than this cut-off were considered influential. We then re-ran the LMMs, excluding samples associated with high-influence animal ID observations, to evaluate the impact of these influential data points on model outcomes.

To test the hypothesis that after antibiotic treatment more socially connected individuals would show closer microbiome resemblance to controls, we first examined whether degree and treatment were drivers of microbiome variation. We used permutational multivariate analysis of variance (PERMANOVA) to test for a global effect of social connectivity and treatment on microbial composition, quantified using four distinct metrics: weighted UniFrac, unweighted UniFrac, Bray–Curtis and Jaccard. We ran separate PERMANOVAs using each community metric as a response variable and degree, treatment status, time, age and interactions between degree × treatment, degree × time and degree × treatment × time as predictor variables. Model permutations were restricted by animal ID to account for repeated sampling of individuals in *vegan* [35].

Next, we used pairwise PERMANOVA models to test for similarities in microbial composition associated with social connectivity and treatment status. To do this, we binned individuals into categories based on degree and treatment. To bin the continuous ‘degree’ variable, we classified individuals with a degree score higher than the population mean plus one standard deviation (>0.02 + 0.013 [0.033]) as having ‘high’ social connectivity and those with degree scores ≤0.033 as having ‘low’ connectivity. We then created four social connectivity–treatment categories: high–treated (*n* = 4 unique individuals, 13 samples), high–control (*n* = 3, 13), low–treated (*n* = 16, 40) and low–control (*n* = 3, 13). Using these categories, we ran all pairwise combinations of PERMANOVAs to test for differences in microbial composition between categories. The pairwise models included the four community metrics (weighted UniFrac, unweighted UniFrac, Bray–Curtis, Jaccard) as separate response variables, with social connectivity–treatment category, age, time and the interaction between social connectivity–treatment category and time as predictor variables. Model permutations were restricted by animal ID to account for repeated sampling of individuals.

Finally, we used differential abundance analyses to identify specific ASVs that contributed to differences observed between control and treated individuals. Differential abundance analyses were performed using Songbird, which calculates log-ratios and then ranks ASVs based on these ratios [36]. We ran separate models for high and low individuals using treated individuals as the reference group. For each model, the predictors included social connectivity–treatment category and time, with a positive log-fold change indicating ASVs enriched in control samples and a negative change indicating ASVs enriched in treated samples. We also performed Wilcoxon tests on the log-ratios of the top-ranked 15 ASVs from control and treated samples within each pairwise comparison to determine whether there were significant differences, which would suggest that the top ASVs were uniquely associated with control or treated individuals within the respective comparison.

## 3. Results

### (a) Social network description

**Figure 1.**
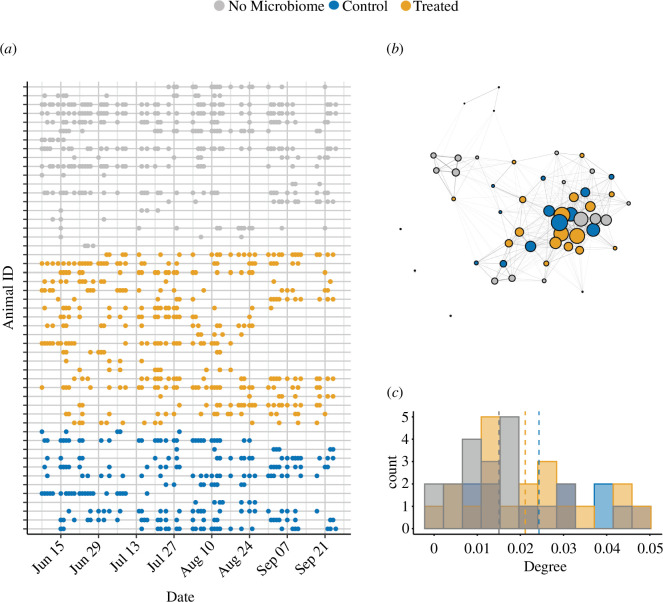
Social network and sampling scheme. A total of 51 female gazelles were tracked to estimate the social network. Of these, 32 individuals were sampled for the microbiome (control (blue): *n* = 12; treated (orange): *n* = 20; no microbiome samples (grey): *n* = 19). (*a*) A plot showing the number of times an individual was re-sighted during the study. (*b*) A network graph showing social connections between individuals, which are represented by nodes. The size of the nodes represents their weighted degree score. The thickness of the lines (edges) connecting individuals represents the number of interactions between individuals (edge weight). (*c*) A histogram showing the distribution of weighted degree scores across individuals by treatment type. Vertical dashed lines show the mean degree for each treatment.

Over the three-month study period, we observed 51 individuals a total of 1028 times (mean = 20.2 ± 12.7 observations per individual, figure 1*a*). Each individual was seen associating with between 1 and 40 (mean = 18.5) unique contacts and the edge density of the social network was 0.340, suggesting a moderate level of connectivity between individuals (figure 1*b*). Weighted degree estimates, which represent the sum of the edge weights for a node, ranged from 0 to 0.048 (mean = 0.02). The degree distribution showed that the network was not highly aggregated (i.e. there is not a pattern of most individuals showing low levels of connectivity and a few showing high levels of connectivity), and importantly, that control and treated individuals were similarly distributed across levels of connectivity (figure 1*c*).

### (b) More socially connected individuals gain microbes faster

**Figure 2.**
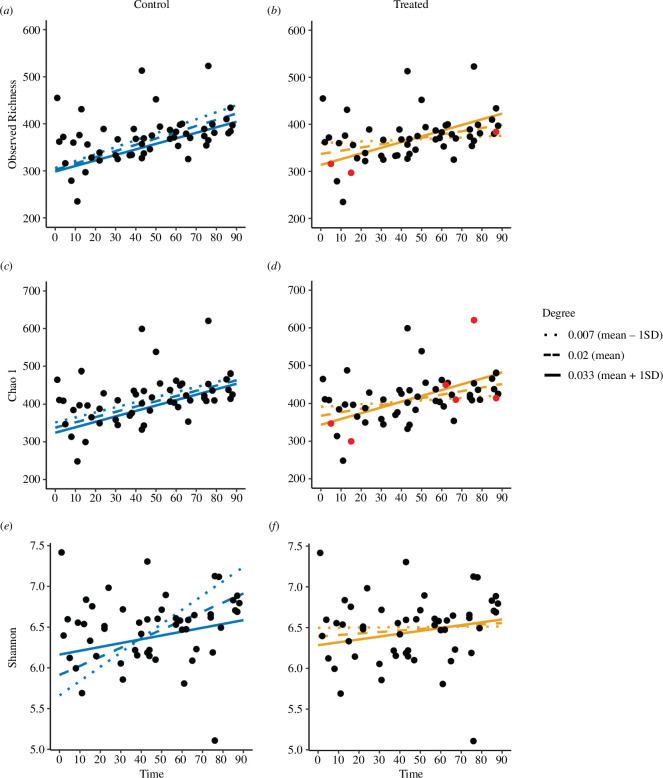
Changes in microbiome alpha diversity over time for different levels of social connectivity, with influential points highlighted. Plots show changes in diversity for control (blue) and treated (orange) individuals measured as (*a,b*) observed richness, (*c,d*) Chao 1 and (*e,f*) Shannon diversity. Lines show model predictions at three different levels of degree (a measure of social connectivity) representing the mean value (0.02, dashed line), 1 standard deviation (s.d.) above the mean (solid line) and 1 s.d. below the mean (dotted line). Red dots show high-influence samples that were excluded in the influence models (electronic supplementary material, table S1).

More socially connected individuals gained microbiome richness faster after antibiotic perturbation. Among antibiotic-treated individuals, but not controls, the interaction between social connectivity and time was a significant predictor of observed richness and a marginally significant predictor of Chao 1 (table 1, figure 2). In both cases, a higher degree was associated with greater increases in microbiome richness over time, suggesting that more socially connected individuals gained microbes more rapidly after treatment (figure 2*a–c*). No such association was observed for Shannon diversity (table 1, figure 2*c*). When we re-ran these two models after removing influential observations, the social connectivity × time effect remained significant for observed richness and disappeared for Chao 1 (electronic supplementary material, table S1).

**Table 1.** Type III ANOVA table based on linear mixed models testing the effect of degree, age, time (days post-treatment) and the interaction between degree and time on observed richness, Chao 1 and Shannon diversity. Model output abbreviations: sums of square (SS), mean squares (MS), degrees of freedom in the numerator (Numd.f.); degrees of freedom in the denominator (Dend.f.). Significant ( ≤ 0.05) and marginal ( ≤ 0.059) *p*-values are given in bold.

**v**ariable	SS	MS	Num**d.f.**	Den**d.f.**	***F***-value	***p*** **‐value**	SS	MS	Num**d.f.**	Den**d.f.**	***F***-value	***p*** **‐value**
**observed richness**												
	**control**						**treated**					
degree	40.2	40.2	1	16.329	0.028	0.869	7195.2	7195.2	1	48	3.646	0.062
age	120.0	120.0	1	7.473	0.084	0.780	1693.9	1693.9	1	48	0.858	0.359
time	3618.2	3618.2	1	32.657	2.524	0.122	138.0	138.0	1	48	0.070	0.793
degree × time	233.8	233.8	1	32.945	0.163	0.689	9572.7	9572.7	1	48	4.850	**0.032**
**Chao 1**												
	**control**						**treated**					
degree	736.95	736.95	1	16.839	0.267	0.612	7585.2	7585.2	1	48	2.331	0.133
age	156.81	156.81	1	6.940	0.057	0.819	3626.1	3626.1	1	48	1.114	0.296
time	2934.47	2934.47	1	30.480	1.062	0.311	2.9	2.9	1	48	0.001	0.976
degree × time	26.62	26.62	1	32.750	0.010	0.922	12682.8	12682.8	1	48	3.897	**0.054**
**Shannon**												
	**control**						**treated**					
degree	0.428	0.428	1	18.273	2.478	0.133	0.149	0.149	1	26.875	0.857	0.363
age	0.002	0.002	1	8.082	0.012	0.914	0.024	0.024	1	16.648	0.138	0.716
time	1.088	1.088	1	23.289	6.302	**0.019**	0.005	0.005	1	47.961	0.031	0.861
degree × time	0.599	0.599	1	29.983	3.471	0.072	0.096	0.096	1	45.154	0.552	0.461

### (c) Microbiomes of more socially connected individuals are more compositionally distinct

**Figure 3.**
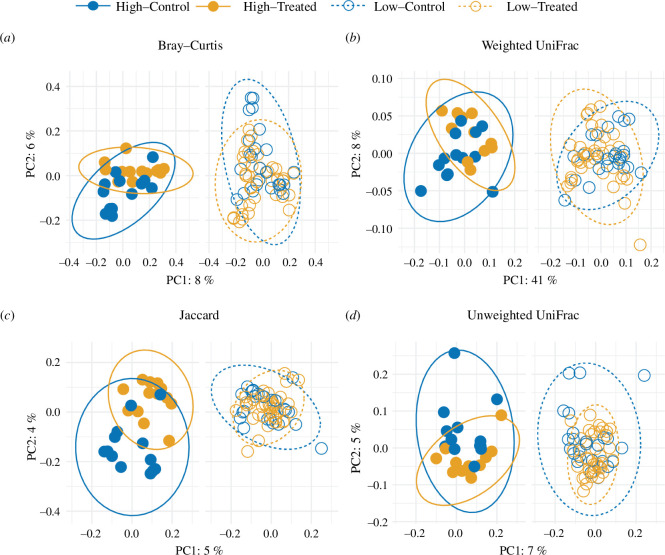
Microbiome communities of high-treated and high-control individuals are more divergent than are communities of low-treated and low-control individuals. Principal coordinate analysis (PCoA) plots showing microbiome differences measured by (*a*) Bray–Curtis, (*b*) weighted UniFrac, (*c*) Jaccard and (*d*) unweighted UniFrac indices. High–treated and high–control individuals showed significant differences in microbiome composition as measured by Bray–Curtis, Jaccard and unweighted UniFrac distances (electronic supplementary material, table S2). Low–treated and low–control individuals showed significant differences only in microbiome composition as measured by Bray–Curtis. Colours represent control (blue) and treated (orange) individuals; closed circles represent high social connectivity and open circles represent low social connectivity. Ellipses show 95% confidence levels for each social category, high (solid lines) and low (dashed lines).

In terms of microbiome composition, following antibiotic treatment, more socially connected individuals were less, not more, similar to their control counterparts. Social connectivity interacted with treatment to explain variation in three of four microbiome community metrics (table 2), suggesting a role for social behaviour in shaping the response to antibiotic treatment. In support, pairwise tests comparing differences in microbiome composition between more versus less socially connected, antibiotic-treated individuals and their control counterparts (high–treated versus high–control and low–treated versus low–control) revealed that for three out of four community metrics (Bray–Curtis, Jaccard, unweighted UniFrac) there were consistent effects of treatment and the interaction between treatment and time on the microbiomes of high–treated compared to high–control individuals (figure 3, electronic supplementary material, table S2). In contrast, the interaction between treatment and time explained variation in microbiome composition for only one of four metrics in the comparison between low–treated and low–control individuals (Bray–Curtis; figure 3, electronic supplementary material, table S2). This difference in responsiveness between lows and highs suggests that following antibiotic treatment, the microbiomes of more socially connected individuals were more likely to diverge from their control counterparts.

**Table 2.** PERMANOVA testing the effect of degree, age, treatment, time and their interactions on microbiome community composition. Model permutations were restricted by animal ID to account for repeated sampling of individuals. Model output term abbreviations are: degrees of freedom (d.f.) and sums of squares (SS). Significant ( ≤0.05) and marginal ( ≤0.059) *p*-values are given in bold.

	d.f.	SS	*R* ^*2*^	*F-value*	*p*‐value
**Bray–Curtis**					
degree	1	0.553	0.027	2.628	**0.037**
age	1	0.390	0.019	1.856	**0.009**
treatment	1	0.333	0.017	1.584	0.057
time	1	0.273	0.014	1.299	0.541
degree × treatment	1	0.427	0.021	2.030	**0.004**
degree × time	1	0.238	0.012	1.130	0.481
treatment × time	1	0.373	0.019	1.774	**0.002**
degree × treatment × time	1	0.271	0.013	1.290	0.491
residual	82	17.249	0.858		
total	90	20.107	1.000		
**Jaccard**					
degree	1	0.368	0.021	2.017	0.068
age	1	0.303	0.018	1.661	**0.034**
treatment	1	0.306	0.018	1.676	0.090
time	1	0.236	0.014	1.290	0.426
degree × treatment	1	0.336	0.020	1.840	**0.059**
degree × time	1	0.220	0.013	1.205	0.124
treatment × time	1	0.222	0.013	1.216	0.268
degree × treatment × time	1	0.261	0.015	1.427	0.066
residual	82	14.969	0.869		
total	90	17.220	1.000		
**unweighted UniFrac**					
degree	1	0.203	0.023	2.206	0.095
age	1	0.148	0.017	1.604	**0.050**
treatment	1	0.181	0.021	1.963	0.111
time	1	0.109	0.013	1.189	0.765
degree × treatment	1	0.173	0.020	1.882	**0.039**
degree × time	1	0.113	0.013	1.233	0.183
treatment × time	1	0.121	0.014	1.314	0.218
degree × treatment × time	1	0.161	0.018	1.749	**0.017**
residual	82	7.540	0.862		
total	90	8.749	1		
**weighted UniFrac**					
degree	1	0.02	0.02	1.89	0.143
age	1	0.02	0.02	1.85	0.147
treatment	1	0.02	0.02	1.79	0.153
time	1	0.01	0.01	0.78	0.876
degree × treatment	1	0.04	0.04	3.51	0.155
degree × time	1	0.01	0.01	0.73	0.578
treatment × time	1	0.05	0.04	3.80	**0.037**
degree × treatment × time	1	0.03	0.03	2.71	0.380
residual	82	0.98	0.83		
total	90	1.18	1.00		

Interestingly, in pairwise comparisons of control individuals with different levels of connectivity (high–control versus low–control) there were no significant effects of social connectivity or the interaction between social connectivity and time on microbiome composition, although there was a trend for one out of four community metrics (Bray–Curtis; electronic supplementary material, table S2). In contrast, social connectivity and/or the interaction between social connectivity and time were strong predictors of variation in microbiome composition across all community metrics in pairwise comparisons between treated individuals with different levels of connectivity (electronic supplementary material, table S2). These patterns suggest that antibiotic treatment magnified social differences in microbiome composition.

### (d) Microbiomes of more socially connected individuals are more taxonomically divergent

**Figure 4.**
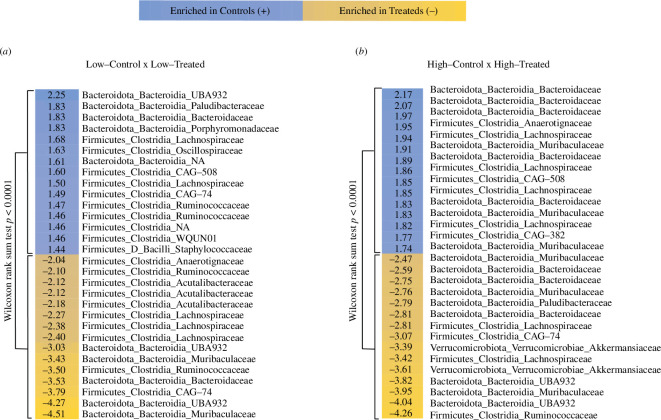
Differential abundance of ASVs in low versus high social connectivity categories in response to antibiotic treatment. (*a*) The top 15 ASVs for the low-connectivity category and (*b*) the top 15 ASVs for the high-connectivity category. For each comparison, positive values indicate ASVs enriched in control individuals (blue) and negative values indicate enrichment in the treated individuals (orange). Rows are labelled with the phylum, class and family of the respective ASV and associated numbers are log-fold differences. *P*-values from Wilcoxon tests quantifying the difference in log-fold change between treatment-enriched ASVs and control-enriched ASVs are shown for each comparison. A list of all differentially abundant ASVs is presented for lows in electronic supplementary material, table S3 and for highs in electronic supplementary material, table S4.

Social connectivity was also associated with differences in microbial taxa enrichment in response to antibiotic treatment. Overall, three taxa emerged as showing differences in enrichment in comparisons between treated and control individuals within social connectivity categories: Bacteroidota, Firmicutes and Verrucomicrobiota. For all comparisons (low–treated versus low–control and high–treated versus high–control), the log-fold change in the top 15 ASVs enriched in control versus treated individuals was significantly different (Wilcoxon tests: low: *W* = 0, *p *< 0.001; high: *W* = 5, *p *< 0.001; figure 4). However, the pattern of taxa enrichment between treated and control individuals was more distinct in the high-connectivity group (figure 4, electronic supplementary material, tables S3 and S4).

Specifically, in the low-connectivity group, 10 of the top 15 ASVs enriched in both control and treated samples were from Firmicutes and five were from Bacteroidota; whereas in the high-connectivity group, control samples were enriched in Bacteroidota (8/15 ASVs) and Firmicutes (7/15), while treated samples were enriched in Bacteroidota (9/15), Firmicutes (4/15) and Verrucomicrobiota (2/15), suggesting a loss of Firmicutes and gain of Verrucomicrobiota in this group.

## 4. Discussion

Microbiome disruptions in wild animals are becoming increasingly common in response to continuous anthropogenic challenges [37]. Understanding mechanisms that maintain microbiome stability and resilience is important for quantifying how species are responding to these challenges. Because social interactions are key drivers of microbiome sharing among group-living animals and may contribute to microbiome resilience, heterogeneity in social behaviour may help explain variation in the resilience of wild animal microbiomes to perturbation [8]. We tested this hypothesis using an antibiotic perturbation experiment in a wild social mammal. We found that high social connectivity led to faster rates of microbiome acquisition after antibiotic disruption. However, the increase in richness resulted in microbiomes that diverged from their control counterparts. These results suggest that higher social connectivity drives rapid rates of microbial accumulation after disruption, but, contrary to expectation, the microbes gained resulted in increased dissimilarity between the microbiomes of disrupted and unmanipulated individuals. Our taxonomic analyses further support this conclusion by revealing greater divergence in specific microbial taxa among more socially connected individuals after treatment.

Rapid increases in species richness after disturbance are a common sign of resilience across ecosystems [38,39]. During ecosystem disruption (e.g. fire, grazing, habitat fragmentation) the creation of new niches can facilitate an increase in species immigration from the regional pool [40–42]. Analogous to large-scale ecosystem disruption, group-level microbiomes can be considered regional pools; thus, the microbiomes of more socially connected individuals within a group should experience higher rates of immigrating microbes during periods of disruption. This has been demonstrated in lab mice, where the microbiomes of cohoused individuals recovered faster than those of singly housed individuals after antibiotic treatment [43]. In support, we found that more socially connected individuals who received an antibiotic treatment gained microbiome richness at a faster rate than less-connected individuals. Specifically, among antibiotic-treated animals, the interaction between social connectivity (measured as weighted degree) and time was a predictor of observed microbial richness and to a lesser degree Chao 1, where individuals with a higher degree increased more quickly in richness over time (figure 2, table 1, electronic supplementary material, table S1). Similarly, studies of chimpanzees, sifakas and woodrats have described positive associations between social connectivity and alpha diversity in a context without antibiotic treatment [2,5,44]. Thus, the more rapid immigration of taxa from the regional (group) microbiome pool into the microbiomes of highly connected individuals in our study, and others, implies that social contact is a key mechanism facilitating the gain in microbial taxa.

Past studies suggest that social transmission of the microbiome tends to increase similarity between group members [3,5,45]. This homogenization process is fundamental to the hypothesis that social contact is a mechanism that facilitates microbiome stability within groups [8]. We performed pairwise analyses using social connectivity–treatment categories (high–control, low–control, high–treated, low–treated) to test for differences in the degree of homogenization between more- versus less-socially connected antibiotic-treated individuals and their respective controls (high–treated versus low–treated and low–treated versus low–control). Counterintuitively, we found that more socially connected individuals that received antibiotic treatment had more distinct microbiomes from their control counterparts when compared to less-connected individuals and their respective controls (figure 3; electronic supplementary material, table S2). This dissimilarity was observed for three out of four measures of microbiome composition: Bray–Curtis, Jaccard and unweighted UniFrac distance. Aligning with these compositional results, our taxonomic analyses showed that high–treated individuals were more taxonomically distinct from their control counterparts than low–treated individuals were from theirs (figure 4).

The compositional dissimilarity observed between high–control and high–treated individuals appeared to result in the replacement of Firmicutes by Verrucomicrobiota in the treated group. Specifically, the enriched Verrucomicrobiota ASVs were from the family Akkermansiaceae, which includes *Akkermansia* spp. that are found in the microbiomes of a wide range of host taxa [46]. Interestingly, *Akkermansia* has been shown to bloom after antibiotic treatment in humans and other animals [47], potentially explaining the enrichment of this taxon in high–treated individuals. However, similar enrichment was absent in the low–treated group and this differential response suggests that social connectivity may be linked to the appearance of Verrucomicrobiota post-antibiotic treatment. Given that *Akkermansia* is known to influence host metabolism [47] and social behaviour can impact resource acquisition [48,49], it is plausible that there are metabolic consequences of the socially mediated differences in microbiome composition we observed. Future research will be needed to understand whether there are functional consequences of these socially mediated microbiome differences.

A surprising observation throughout our study was that social connectivity was only weakly associated with microbiome responses in individuals that did not receive antibiotic treatment. For measures of both alpha diversity (observed richness and Chao 1) and beta diversity (Jaccard and unweighted UniFrac), antibiotic perturbation magnified social effects on the gazelle microbiome. One reason why antibiotic perturbation may have been key to revealing the role of social behaviour is the environmental context of our study. Specifically, during our three-month study period, both rainfall and vegetation greenness declined sharply at the study site [21]. Importantly, the changes in vegetation greenness were associated with variation in microbiome diversity in gazelles [21], likely a reflection of rainfall-driven dietary changes [21,50,51]. Consequently, this backdrop of strong environmentally mediated change in the microbiome could have muted socially mediated effects. In support of this idea, Archie & Tung [7] recommended that perturbation experiments, which shift the microbiome from its equilibrium state, are necessary to reveal differences owing to social interactions. Yet, to date, most wild animal social behaviour–microbiome studies have been done under conditions where the microbiome is in an ‘equilibrium’ or non-perturbed state [2–6], which could mask the type of counterintuitive patterns we describe here. More generally, given that environmental dynamism is a common feature of studying natural populations, our results reinforce the value of using a perturbation approach to understand social behaviour–microbiome interactions in the wild.

Overall, our study reveals new insight into the complexities of how social behaviour influences microbiome composition. It has been suggested that antibiotic treatment experiments are necessary to fully understand how social contact shapes the microbiome [7]. Our study provides strong support for this suggestion. Under antibiotic treatment conditions, we found that high levels of social connectivity facilitated the rapid acquisition of microbes after disruption, but this resulted in greater microbiome divergence from the population background (i.e. control) microbiome. The microbiome dissimilarity observed between treated and control individuals with high connectivity may reflect a change in host–microbiome function in more socially connected individuals. Longer- term experiments are required to quantify whether the divergent microbiomes observed in more social individuals have negative or positive consequences for the host. Such experiments could be aided by recent advances in wildlife tracking technologies (e.g. lightweight proximity and GPS tags [52,53]) that would facilitate the generation of higher resolution social networks. Regardless, our finding that social connectivity can increase microbiome dissimilarity, rather than similarity, in the context of a perturbation event raises new questions about the costs and benefits of social interactions.

## Data Availability

Raw sequence data are available in the European Nucleotide Archive (Project: PRJEB14530) [54]. Code, processed data and metadata can be found on Dryad [55]. Supplementary material is available online [56].
